# iTRAQ-based proteomic analysis reveals the mechanisms of *Botrytis cinerea* controlled with Wuyiencin

**DOI:** 10.1186/s12866-019-1675-4

**Published:** 2019-12-11

**Authors:** Liming Shi, Beibei Ge, Jinzi Wang, Binghua Liu, Jinjin Ma, Qiuhe Wei, Kecheng Zhang

**Affiliations:** 1 State Key Laboratory of Biology of Plant Diseases and Insect Pests, Institute of Plant Protection, Chinese Academy of Agricultural Sciences, Beijing, People’s Republic of China; 2 Guangxi Key Laboratory of Utilization of Microbial and Botanical Resources, Guangxi Key Laboratory for Polysaccharide Materials and Modifications, School of Marine Sciences and Biotechnology, Guangxi University for Nationalities, Nanning, People’s Republic of China

**Keywords:** *Botrytis cinerea*, Wuyiencin, Proteomic, Isobaric tags for relative or absolute quantitation (iTRAQ), Parallel reaction monitoring (PRM)

## Abstract

**Background:**

Grey mould is an important plant disease worldwide, caused by *Botrytis cinerea,* resulting in serious economic loss. Wuyiencin, a low toxicity, high efficiency, and broad-spectrum agricultural antibiotic, has been demonstrated effectiveness against *B. cinerea.*

**Results:**

Wuyiencin treatment inhibited growth and sporulation of *B. cinerea,* specifically altering hypha morphology and intracellular structures. These changes were accompanied by differential expression (fold change > 2.0) of 316 proteins identified by iTRAQ-labelling LC-MS/MS analysis (*P* < 0.05). Up-regulation of 14 proteins, including carbohydrate metabolism proteins and cell wall stabilization proteins, was validated by parallel reaction monitoring (PRM). Down-regulation of 13 proteins was validated by PRM, including regulators of energy metabolism, nucleotide/protein synthesis, and the biosynthesis of mediators of plant stress and decay.

**Conclusion:**

Our results confirm the inhibitory biological effects of wuyiencin on *B. cinereal* and elaborate on the differentially expressed proteins and associated pathways implicated in the capacity of wuyiencin to debilitate the growth and pathogenicity of grey mould. This study provides validated candidates for further targeted exploration with the goal of optimizing wuyiencin as a safe, low-toxicity agent for biological control.

## Background

Grey mould is a fungus known scientifically as *Botrytis cinerea*. It is a common cause of disease among over 200 plant species, including fruit and vegetable plants, and flowers. Because *B. cinerea* is widely distributed through the air, it can spread quickly causing widespread pathogenesis and major economic losses in agriculture [1]. Currently, *B. cinerea* control is largely achieved by chemical measures. Popular Botryticides include fludioxonil, tebuconazole, iprodione, boscalid, and benzimidazole. In general, these and other agricultural antimicrobial agents function by destabilizing the cell membrane and inhibiting protein and nucleic acid synthesis as well as cellular respiration; however, resistance to these antagonistic properties is not uncommon. The continuous application of fungicides is associated with not only the challenge of high reproductive speed and wide genetic variation of *Botrytis*, but also adaptation that can lead to the development of resistance [2, 3].

The extensive use of chemical fungicides has prompted recent concern regarding the quality of crops, food and human safety, as well as its contribution to the ecological footprint. In short, as the demand grows for chemical-free vegetables and fruits, there remains a need to produce high-efficiency, low toxicity and environmentally friendly solutions to deal with necrotrophic fungi, such as *B. cinerea*. Wuyiencin is produced by *Streptomyces albulus* var. *wuyiensis* [4] and is widely used as an antifungal agent in agriculture [5]. It has demonstrated success in combating fungal diseases among various vegetables and fruit trees [6]. Compared with traditional chemical pesticides, wuyiencin has demonstrated greater environmental compatibility, is non-toxic to humans and animals, does not pollute the environment, and leaves no residues [7]. Wuyiencin has exhibited antimicrobial properties against organisms such as *Botrytis cinerea*, *Rhodotorula rubra*, *Bacillus subtilis*, *Bacillus megaterium*, *Escherichia coli*, *Cladosporium fulvum* and *Staphylococcus aureus* [8].

Although wuyiencin can well control grey mould (*B. cinerea*), up till now, few studies on the mechanism of wuyiencin in controlling *B. cinerea* have been reported. Of the work that has been published, one seminal study demonstrated that wuyiencin is effective in inhibiting *Botrytis* spore germination and to a lesser degree, mycelia protein production. Additional traits that have been characterized in infected tomato seedlings include its ability to alter cell membrane permeability, induce vacuole formation and reduce hypha pathogenicity. This study also identified important enzymatic regulators triggered by the hosts as a defensive response to *B. cinerea* infection [9]. In this study, the time- and concentration-dependent effects of wuyiencin treatment of *B. cinerea*-infected tomato plants were also characterized. This study revealed a significant reduction in plant fungal density 5 days after infection, although fungicidal properties were observed as early as 3 days after infection. Wuyiencin treatment protected the plant and eliminated symptoms in the long-term, maintaining fungal densities at almost zero [7]. The anti-fungal properties of wuyiencin, specifically against *B. cinerea*, support the observations of Mukherjee and Sen, who independently noted that extracellular hydrolytic enzymes may assist *Streptomyces*-derived wuyiencin in the degradation of pathogenic fungi [10].

In the era of high-throughput molecular techniques, the full genomic sequencing of the standard *B. cinerea* strain, B05.10, set the stage for large-scale studies of disease-associated and antifungal conferring genes and gene products of uncharacterized and novel agents for biological control. Previous evidence has shed light on the key players involved in the pathogenic signaling of *Botrytis*-infected plants. For example, the deletion of various cAMP signaling genes has been shown to slow growth of B05.10 and reduce pathogenicity [11]. Similarly, the deletion of the MAP kinase *BMP1* gene slows the growth rate and reduces sporulation and pathogenicity of this strain [12]. Other targets, like the *BcSAK1* and *BMP3* genes, regulate virulence, conidia production, and host-pathogen interactions [13, 14].

Proteomics studies of antibacterial and antifungal agents have focused predominantly on medical applications, with few studies of agricultural pathogens or their regulators. Previous studies clearly demonstrate that microbiological control agents predominantly function through their impact on the proteome. Many antiseptics act against pathogenic microorganisms through the direct binding of bacterial ribosomes to inhibit protein synthesis, the induction of endoplasmic reticulum stress, and the activation of autophagic mechanisms of the host [15]. It is important to elucidate the involvement of these same mechanisms in the action of agricultural control agents [16].

The overall aim of this study was to examine the action of wuyiencin on *B. cinerea* from a top-down approach. Using morphological and subcellular assessments, we first characterized the role of wuyiencin in *B. cinerea*. Next, high-throughput proteomic analysis was carried out to identify differentially expressed proteins and protein networks to further characterize the biological actions of wuyiencin and shed light on the susceptibility of *B. cinerea*.

## Results

### Effects of wuyiencin on the growth and physiological status of *Botrytis cinerea*

**Fig. 1:**
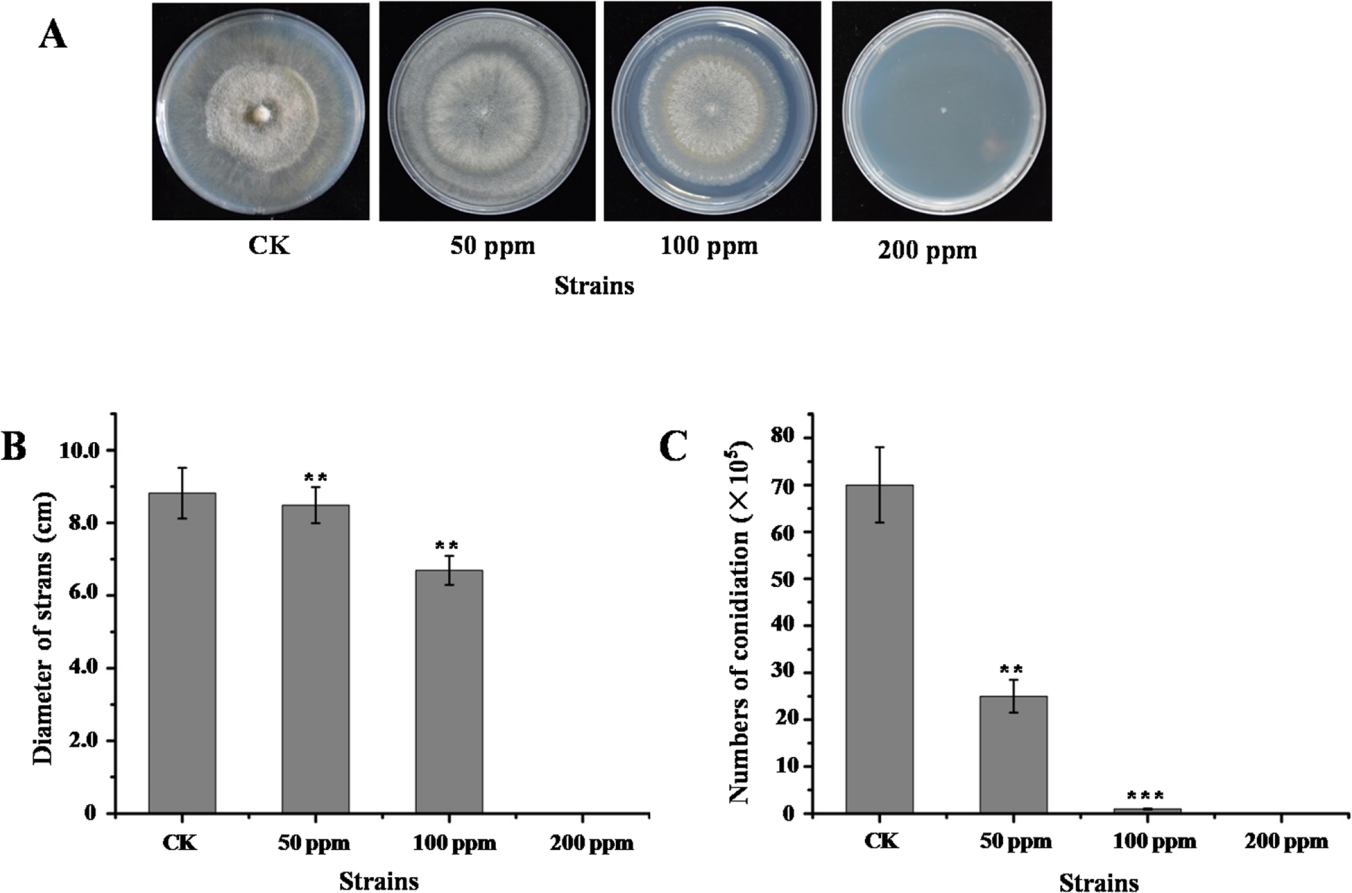
Growth and morphology of wuyiencin-treated *B. cinerea.* **a**. Phenotypes on PDA. Photo was taken at day 7. **b**. The growth rate of *B. cinerea* is decreased by 3.7, 23.9 and 100.0% by treatment with wuyiencin at 50 ppm, 100 ppm and 200 ppm, respectively. **c**. Sporulation is similarly decreased by 64.3, 98.6 and 100.0% by treatment with wuyiencin at 50 ppm, 100 ppm and 200 ppm, respectively. Values are means ± S.E.M of three independent experiments. **P < 0.01 and ***P < 0.001, determined by Student’s *t* test

The standard *B. cinerea* strain B05.10 was inoculated onto PDA medium containing wuyiencin at final concentrations of 0 ppm, 50 ppm, 100 ppm and 200 ppm, and incubated at 20 °C. Mycelial morphology was observed under a light microscope on day 3, while conidia were eluted (0.2% Tween20) and quantified on day 7. The results showed that treatment with progressively higher concentrations of wuyiencin impeded the growth and development of *B. cinerea*, including a reduction in the presence of pigmented hyphae (Fig. 1a, b) and the number of spores detected (Fig. 1c). Specifically, *B. cinerea* growth rate was decreased by 3.7, 23.9 and 100.0% and sporulation was decreased by 64.3, 98.6 and 100.0% under treatment with wuyiencin at 50 ppm, 100 ppm and 200 ppm, respectively.

**Fig. 2:**
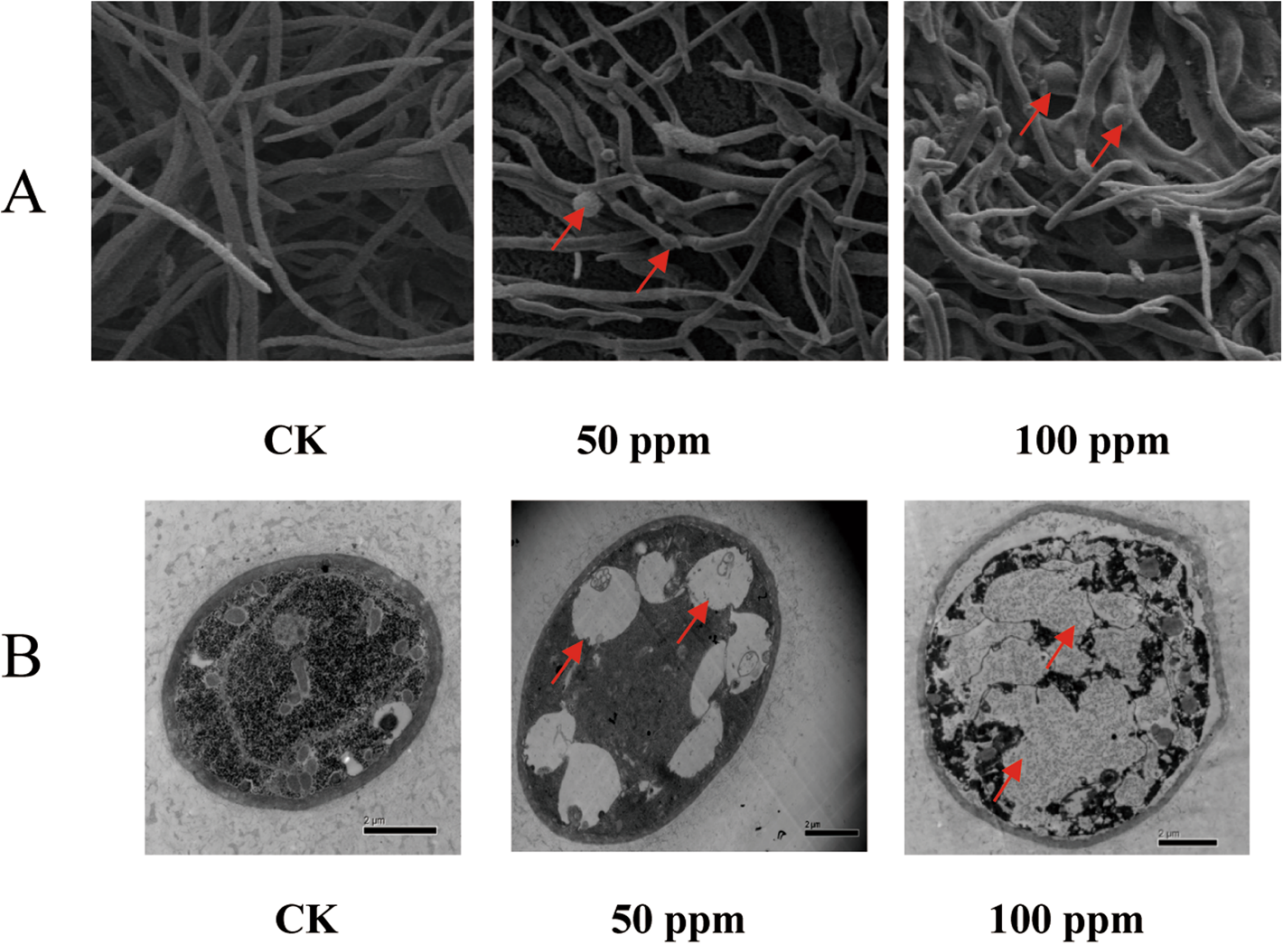
Transmission electron microscopy observations of *B. cinerea* hyphae treated with 0 ppm, 50 ppm and 100 ppm wuyiencin for 7 days. **a**. Compared to control hyphae, wuyiencin-treated hyphae are diminished in number and increased thickness. The appearance of globular anomalies are also apparent only in wuyiencin-treated hyphae (red arrows). SU8010 10.0 kV 8.0 mm × 200 LM (UL). **b**. Compared to untreated *B. cinerea* hyphae, large vacuoles are apparent in wuyiencin-treated specimens (red arrows) .The scale bar indicates 2 μm

After 7 days, TEM demonstrated that the hyphae of *B. cinerea* were deformed. Specifically, the thickness of the hyphae was markedly increased and the branch number was reduced in a dose-dependent manner. Interestingly, globular vesicle formation was apparent following wuyiencin treatment in a dose-dependent manner (Fig. 2a). Ultrastructurally, the number of organelles within the hyphae was decreased by wuyiencin treatment, with the formation of some central large vacuoles inside some wuyiencin-treated hyphae (Fig. 2b).

### Proteomic evaluation of wuyiencin-treated *Botrytis cinerea*

**Fig. 3:**
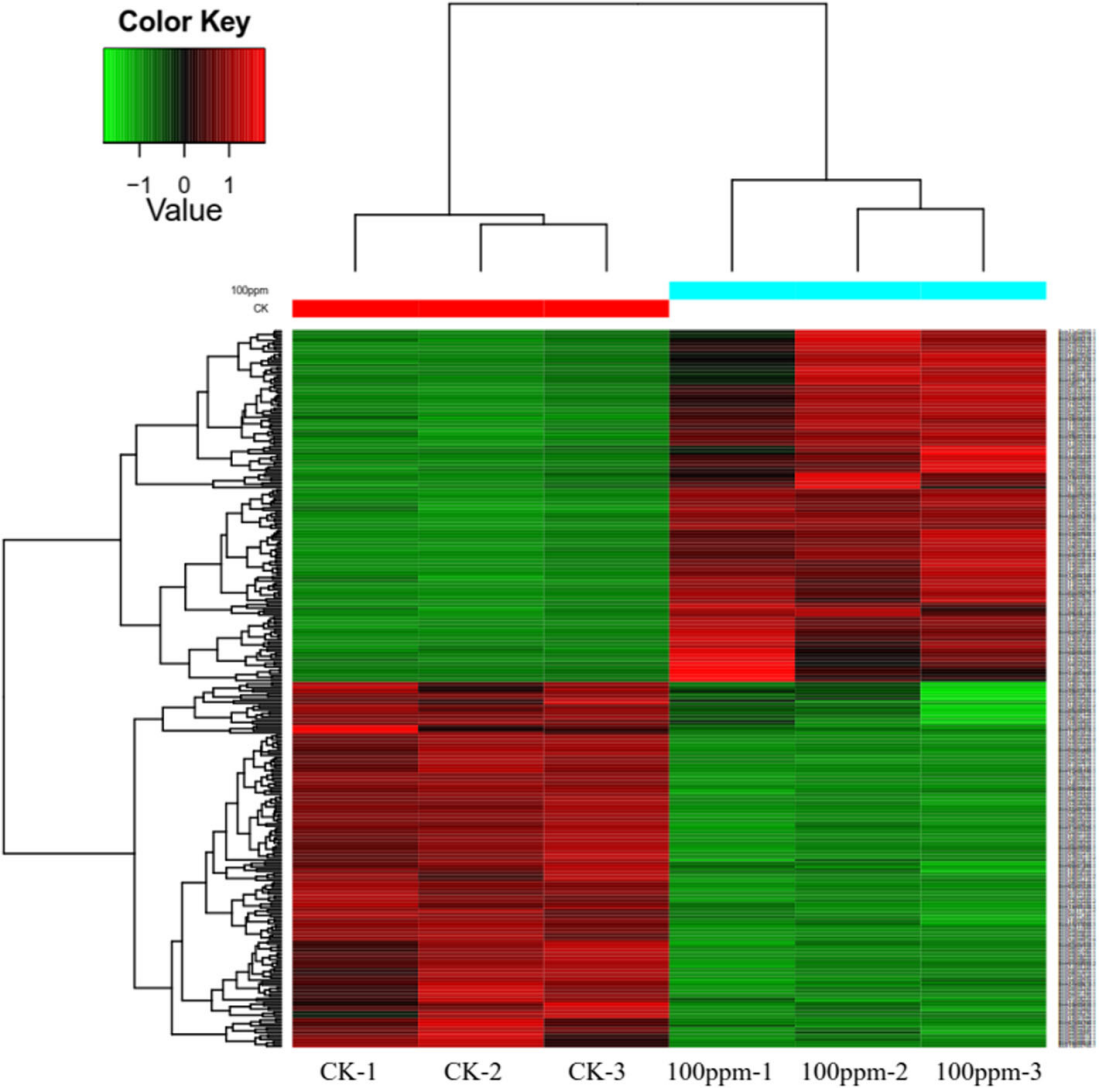
Differential protein cluster diagram of *B. cinerea* treated with 100 ppm wuyiencin for 7 days. The 316 differential proteins identified in the *B. cinerea* dataset were clustered using the multi-sample expression pattern cluster analysis to determine the differentially expressed proteins in comparisons between the control and wuyiencin-treated samples. Each row in the diagram represents a protein, each column is a sample/repeat (the log2 value of the quantitative value is plotted and median correction was performed). CK: control; 100 ppm: wuyiencin-treated

Overall, 22,215 peptides corresponding to 4155 protein groups were identified (marking efficiency 98.12%) by iTRAQ-labelling LC-MS/MS analysis. Differential protein screening (determined by the ratio in the treated samples and their corresponding untreated controls) was performed under the condition of 2.0 difference multiples (fc = fold change) and *P* < 0.05 threshold. Under these conditions, 3816 common proteins were identified, of which 155 proteins were up-regulated and 161 were down-regulated (*P* < 0.05). Hierarchical cluster analysis showed that the three replicates of each condition were clustered into the same respective clades, indicating the reproducibility and reliability of the LC-MS/MS data. Dendrograms further revealed clustering of proteins that were either up-regulated (red) or down-regulated (green) (Fig. 3).

**Fig. 4:**
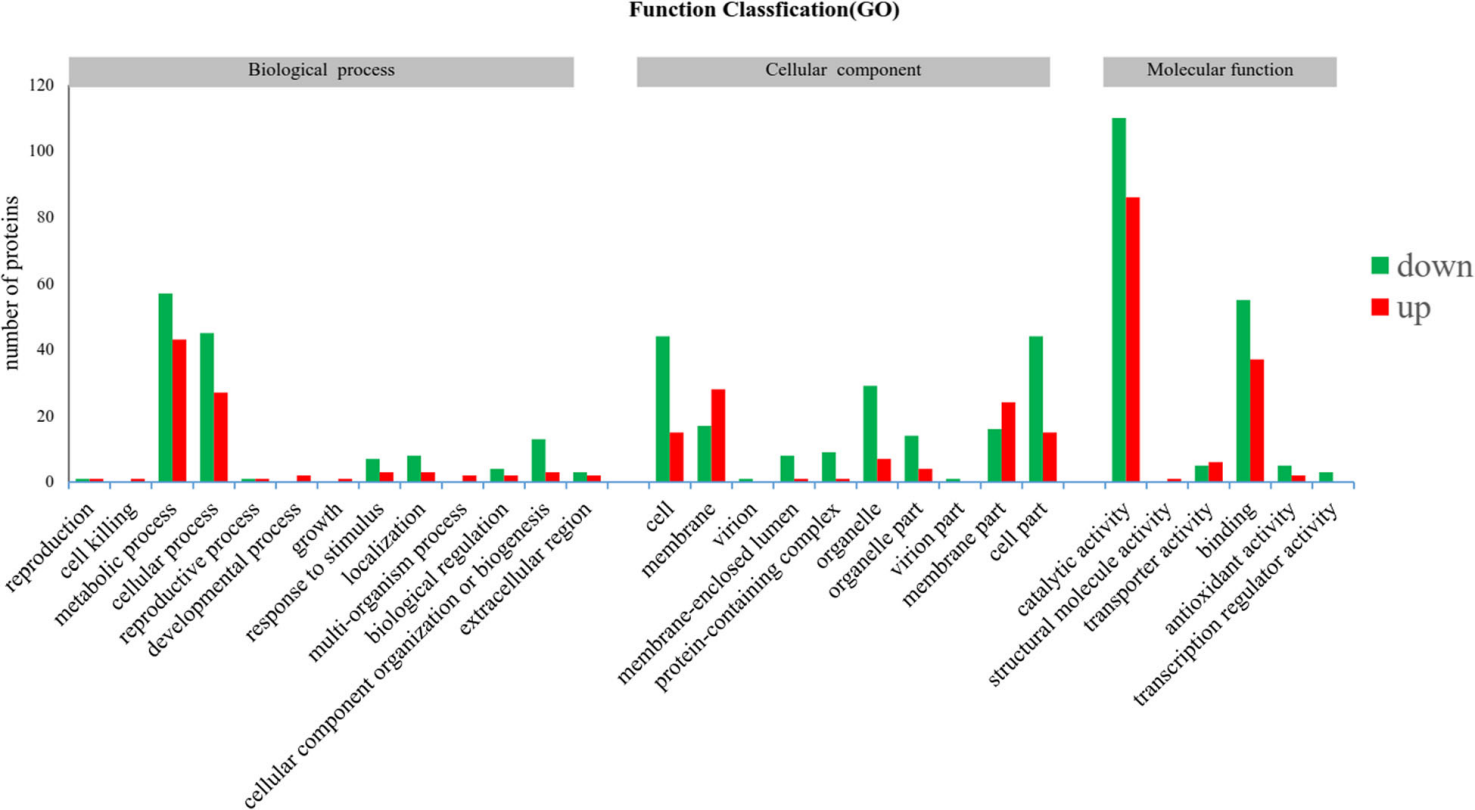
GO classification of differentially expressed proteins of *B. cinerea* treated with 100 ppm wuyiencin for 7 days. The y- axis is a representation of the number of differentially expressed proteins identified in this study, and the x-axis represents the GO classification description of the differentially expressed proteins. Biological Process: all classification descriptions related to biological processes in GO classification; Cellular Component: all categories related to cell composition in GO classification; Molecular Function: all classification descriptions of proteins related to molecular function in GO classification

In total, 316 differentially expressed proteins were assigned GO annotations. The most differentially expressed proteins were assigned to either the cellular or metabolic processes subcategories. These proteins were predominantly located in the cytosol, cell membrane, or in organelles and overwhelmingly participated in catalytic reactions or protein binding (Fig. 4). Interestingly, cytosolic and organelle localized proteins tended to be down-regulated by wuyiencin, while membrane proteins were largely up-regulated.

**Fig. 5:**
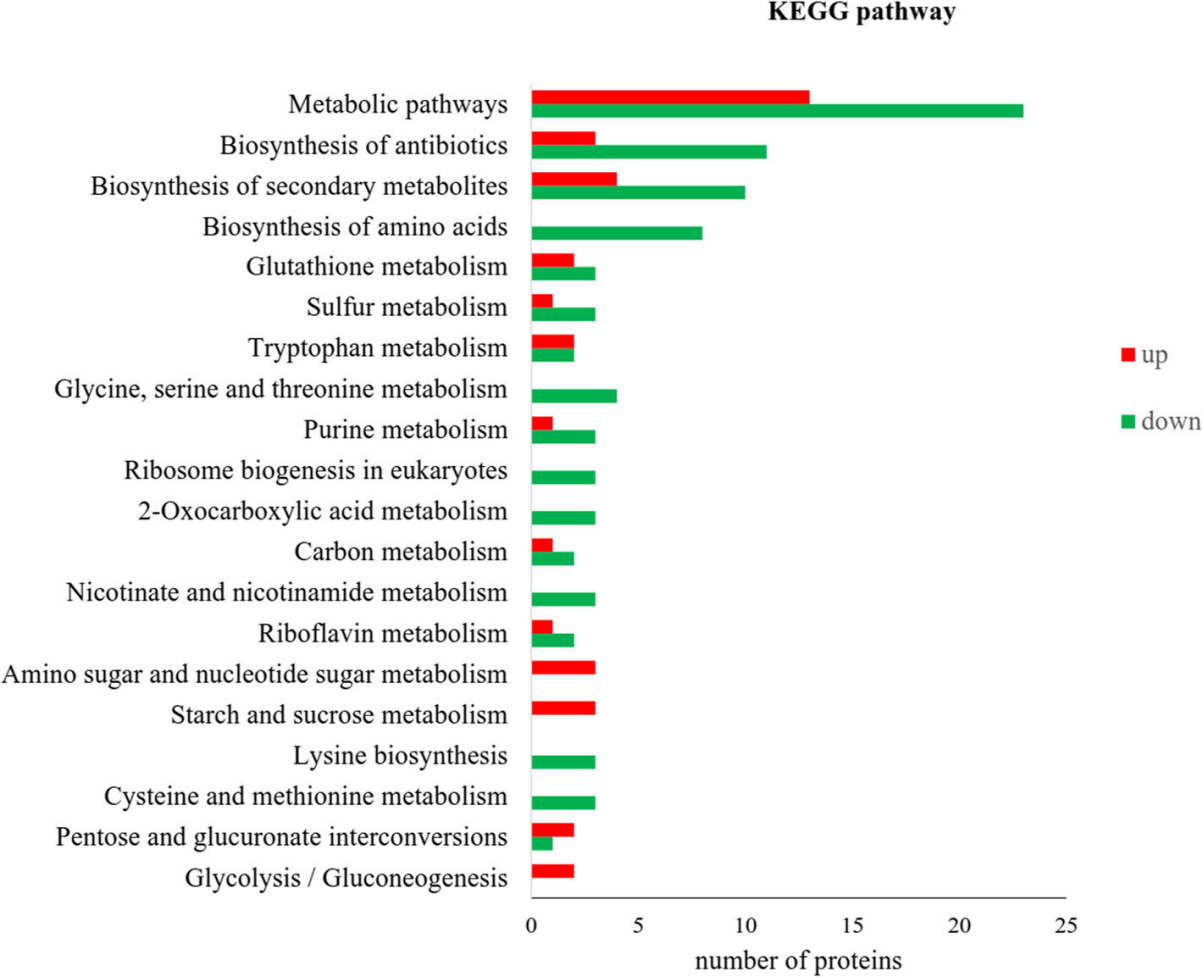
KEGG classification of differentially expressed proteins of *B. cinerea* treated with 100 ppm wuyiencin for 7 days. The x-axis denotes the number of differentially expressed proteins identified; the x-axis indicates the description of each KEGG pathway identified

KEGG functional annotation of differentially expressed proteins showed enrichment across general metabolic pathways, biosynthesis of antibiotics, and biosynthesis of secondary metabolites (with > 10 proteins differentially expressed in each category). Pathways that were almost exclusively up-regulated by wuyiencin treatment included amino sugar and nucleotide sugar metabolism, starch and sucrose metabolism, and glycolysis/glucogenesis proteins. On the other hand, pathways that were almost exclusively down-regulated by wuyiencin included amino acid biosynthesis and metabolism, ribosome biogenesis, nicotinate and nicotinamide metabolism, and 2-oxocarboxylic acid metabolism proteins (Fig. 5). These results indicated the importance of a variety of sugar-based metabolic and protein synthesis groups for fungal energy/growth and the regulation of the ability of fungi to degrade plant biomass.

Finally, PRM mass spectrometry was performed to validate the quantification results obtained by discovery-based proteomics analysis. Based on pre-experimental detection and screening, 487 peptide fragments were used to quantify 50 differentially expressed proteins. Using this technique, incongruent results were obtained for 11 targeted proteins compared to those obtained in the discovery-based proteomic analysis. Of the 39 differentially expressed proteins with consistent results, the differences were found to be statistically significant for 27. Fourteen proteins were up-regulated (fold change 1.4–7.1) by wuyiencin treatment, while 13 were down-regulated (fold change 0.6–0.08) (Table 1). Up-regulated proteins validated by PRM were predominantly hydrolytic, playing roles in carbohydrate metabolism, cell wall stabilization, protein synthesis and phospholipid biosynthesis. The majority of down-regulated proteins displayed oxidoreductase activity, playing roles in energy regulation, protein/amino acid biosynthesis and nucleic acid metabolism. The PRM validated proteins implicated similar KEGG annotated pathways compared to those implicated by the discovery-based proteomic analysis.

**Table 1:** PRM validated differentially expressed proteins in wuyiencin-treated *B. cinerea*

Accession No.	Description	Molecular Function	Biological Process	iTRAQ		PRM	
				FC	*p*-Value	FC	*p*-Value
Bcin03p07210.1	Putative alpha beta hydrolase fold protein	Hydrolase activity	Metabolic process	4.862	0.001	7.118	0.035
Bcin01p10150.1	Similar to phosphatidylserine decarboxylase	Phosphatidylserine decarboxylase activity	Phospholipid biosynthetic process	3.621	0.004	4.352	0.004
Bcin10p00030.1	Putative dimeric alpha-beta barrel protein	Secretase and lipase activity	Virulence/adaptation	3.450	0.003	4.317	0.007
Bcin12p02040.1	Similar to aspartic protease (Secreted protein)	Inactive proenzyme (pepsin family)	Cell wall stabilization	2.850	0.011	3.885	0.041
Bcin12p06180.1	Cyanide hydratase	Cyanide hydratase activity, hydrolase activity (acting on carbon-nitrogen but not peptide bonds)	Cyanide catabolic process	3.004	0.006	3.422	0.016
Bcin15p00520.1	Esterase	Hydrolysis enzyme	Cell membrane permeabilization	3.514	0.007	2.938	0.005
Bcin17p00040.1	Putative prolyl aminopeptidase protein	Aminopeptidase activity	Protein synthesis, assembly, fate and degradation	2.637	0.001	2.853	0.013
Bcin01p10140.1	Similar to flavin-nucleotide-binding protein	Co-factor binding; hydrolase activity	Oxidative metabolism	4.776	0.013	2.593	0.016
Bcin04p01400.1	Similar to short-chain dehydrogenase/reductase sdr	Oxidoreductase activity	Integral membrane protein	2.909	0.001	2.537	0.031
Bcin11p02720.1	Putative glycerol dehydrogenase protein	Oxidoreductase activity	Metal ion binding (NAD,zinc)	3.317	0.001	2.489	0.004
Bcin06p00620.1	Putative tripeptidyl peptidase a protein	Serine-type endopepidase activity	Metal ion binding (Ca^2+^ cofactor)	4.850	0.019	2.389	0.022
Bcin07p04810.2	Beta-hexosaminidase	Hydrolysis enzyme	Carbohydrate metabolism	2.668	0.027	1.838	0.015
Bcin12p03390.1	Glucoamylase	Glucan 1,4-alpha-glucosidase activity	Polysaccharide catabolic process	3.160	0.001	1.703	0.043
Bcin01p08110.1	Alpha-galactosidase	Lysosomal enzyme	Carbohydrate metabolism	2.651	0.038	1.418	0.004
Bcin10p01350.1	Similar to short-chain dehydrogenase/reductase SDR	Oxidore ductase activity	Integral membrane protein	0.195	0.001	0.087	0.004
Bcin03p06060.1	Inosine triphosphate pyrophosphatase	Metal ion binding; nucleoside riphosphate diphosphate activity, NADH pyrophosphatase activity, nucleotide binding	Deoxyribonudeoside triphosphate catabolic process; nucleotide meabolism	0.269	0.001	0.119	0.005
Bcin02p04380.1	Putative monooxygenase fad-binding protein	FAD; monooxygenase activity	ABA biosynthesis	0.169	0.003	0.123	0.004
Bcin13p01010.1	Similar to aflatoxin biosynthesis ketoreductase nor-1	Oxidoreductase activity	Aflatoxin biosynthesis	0.172	0.001	0.161	0.004
Bcin04p05700.1	Putative nadp-dependent alcohol dehydrogenase protein	Alcohol dehydrogenase (NADP+) activity; zinc ion binding	NADP/NADPH balance	0.328	0.007	0.174	0.026
Bcin06p00530.1	Putative nadp-dependent l-serine l-allo-threonine dehydrogenase ydfg protein	Oxidoreductase activity	Amino acid catabolism and transport	0.201	0.001	0.180	0.009
Bcin06p07160.1	Putative peptidase s58 protein	Serine peptidase	Cell cycle regulation	0.185	0.001	0.187	0.001
Bcin06p01180.1	Putative catalase isozyme p protein	Catalase-peroxidase activity	Oxidative stress response	0.214	0.006	0.222	0.027
Bcin10p05150.1	Eukaryotic translation initiation factor 6	Free ribosomal binding	Inhibits ribosomal subunit binding; inhibits cell growth	0.268	0.001	0.291	0.042
Bcin03p09280.1	Putative saccharopine dehydrogenase protein	Oxidoreductase activity	Lysine biosynthesis	0.226	0.001	0.305	0.016
Bcin03p04480.1	Similar to oxidoreductase	Oxidoreductase activity	Stress adaptation	0.235	0.004	0.430	0.031
Bcin03p00400.1	Similar to GNAT family acetyltransferase	Heme binding; oxidoreductase oxygen binding	FAD/NAD(P)-binding domain protein	0.208	0.001	0.461	0.037
Bcin14p00610.5	Endopolygalacturonase 2	Polygalacturonase activity	Carbohydrate metabolism; cell wall organization	0.150	0.001	0.653	0.034

Accession no. is the locus name of a gene in *Botrytis cinerea* genome. *FC* fold change (the protein abundance 100 ppm/ck); *NAD* nicotinamide adenine dinucleotide; *ABA* abscisic acid; *FAD* flavin adenine dinucleotide; *SDR* short-chain dehydrogenase/reductase; *NADP* nicotinamide adenine dinucleotide phosphate; *GNAT*: Gcn5-related N-acetyltransferases. All proteins *P* < 0.05.

## Discussion

The biological mechanisms by which wuyiencin inhibits pathogenic fungi remains to be fully elucidated. While widely used in China as an effective fungicide and already optimized from a production standpoint [17], little is known about the molecular drivers of the fungicidal action of wuyiencin. In this study, we observed morphological and ultrastructural characteristics of wuyiencin-treated *B. cinerea* in line with earlier reports [9]. Importantly, our novel proteomic screening and evaluation revealed candidate pathways and targets that may offer insights into the protective and antagonistic mechanisms underlying the effects of this biological control agent.

Some of the earliest insights into the *Botrytis*-wuyiencin relationship included a morphological characterization provided by Sun et al. [9], in which mycelia protein production was significantly reduced, and germination of conidia spores was almost completely inhibited by wuyiencin. In our study, the fungal/mycelia density was drastically reduced in a dose-dependent manner. Similar to the results of Sun et al. [9], 100 ppm wuyiencin was sufficient to completely inhibit spore production. These effects are likely to underlie the impact of wuyiencin on the survival and propagation of *B. cinerea* at the published concentrations.

Previous insights into the changes in morphology of *B. cinerea* that occur in the presence of wuyiencin have also been provided by TEM studies [9]. In the present study, ultrastructural evaluation demonstrated hypha deformity, with significantly reduced branching, and increased thickness of existing branches. This is meaningful as the preservation of hypha morphology is essential for the nutrition and survival of fungi. Interestingly, an ultrastructural study also showed hypha abnormalities as well as enlargement of conidial tubes [9], although the latter was not an observation supported by our study. In contrast, our TEM observations revealed additional hypha abnormalities in the form of globular structures formed at the hypha apex. This swelling may be indicative of abnormalities in the calcium/ion signaling processes which regulate vesicular fusion during hypha growth [18]. Interestingly, the antifungal effects of a bacteria-derived culture filtrate against the organism *Fusarium graminearum* exhibited similarly severe hypha swelling [19]. Based on the stringent PRM validation criteria in the present study, the significant upregulation of the Ca^2+^ binding protein (putative tripeptidyl peptidase a protein) (Table 1) could be contributing to hyphal swelling in *Botrytis*. It is important to note, however, that the localized Ca^2+^ gradient in fungi is maintained by a variety of mechanisms including vesicle fusion, cell wall synthesis, osmotic dynamics, etc., and is not clearly understood in fungi [18].

Another discrepancy between the previous TEM observations and our own was the detection of membrane structure. TEM observations showed that the membrane structure was compromised at high concentrations of wuyiencin and provided details of membranous insult which lead to cellular leakage [9]. However, the cell wall is not a one-dimensional structure in fungi, and hydrolases at the cell wall are known to play a role as cell wall remodeling enzymes [20]. The secondary layer detected in our stress condition could signal cell wall architectural remodeling based on the rearrangement of stratified cell wall materials. This phenomenon, though not observed in Sun et al.’s study has been observed during sporulation [20] and in aged fungal walls, though some evidence exists that secondary layers can also result from chemical treatment and other stressors [21]. Similar to this findings, we observed the appearance of enlarged vacuolar structures in wuyiencin-treated *B. cinerea*. Vacuoles in filamentous fungi are largely interconnected with the growth and morphology of the organism. It has been proposed that vacuolar enlargement may occur to reduce the metabolic demands of the cytosol during periods of nutrient deficits [22]. Additionally, others have characterized large vacuoles as the primary carriers of acid phosphatases and as frequent purveyors of autophagocytosis [23]. Overall, our morphological and ultrastructural characterization of wuyiencin-treated *B. cinerea* supports earlier studies indicating the antagonistic potential of wuyiencin on the overall growth, spore formation, hypha development, and the appearance of various subcellular anomalies.

Proteomics is a valuable tool for the identification of variable proteins associated with critical *B. cinerea* cues including growth, sporulation, and virulence. Proteomic analysis of has not been previously undertaken. In this study, the high-throughput proteomic analysis of *B. cinerea* from the perspective of wuyiencin treatment was performed. Tandem labelling mass spectrometry and bioinformatics analyses was used to identify the main protein networks and hundreds of differentially expressed *B. cinerea* proteins. A significant group of the PRM validated, up-regulated proteins identified in this study are known to exhibit hydrolytic activity. Fungal hydrolases aid a wide range of functions from the breakdown of plant cell walls, to polymer breakdown and nutrient capture [24]. Other hydrolases play a role in the structure of the fungal cell wall itself [25, 26], including regulation of cell wall synthesis and hydrolysis at the hyphal apex, particularly in filamentous fungi [27]. On the other hand, almost half of the PRM validated, down-regulated proteins identified in this study display oxidoreductase activity. While fungal oxidoreductases play a role in a wide array of processes, several are known to be involved in biomass degradation, mediating electron transfer for cellular respiration, and even participating in the generation of free radicals [28]. As shown in Table 1, our evaluation of the general *Botrytis* pathways impacted by wuyiencin treatment showed that although a variety of proteins and processes are affected, carbohydrate metabolism associated proteins were significantly up-regulated, while protein, amino acid, and nucleotide biosynthesis proteins were down-regulated. These observations may, on a broad level, indicate nutritive restriction in *B. cinerea*, wherein stores of starch, saccharides and other glycol-conjugates are hydrolyzed to supplement as carbon sources. This would align with the observed decrease in amino acid biosynthesis, a phenomenon observed during starvation of pathogenic fungi [29]. The essential role of amino acids in the maintenance of central metabolism, and the stimulation of morphogenesis and virulence are also consistent with the early characterizations of wuyiencin-treated *B. cinerea* [30].

**Fig. 6:**
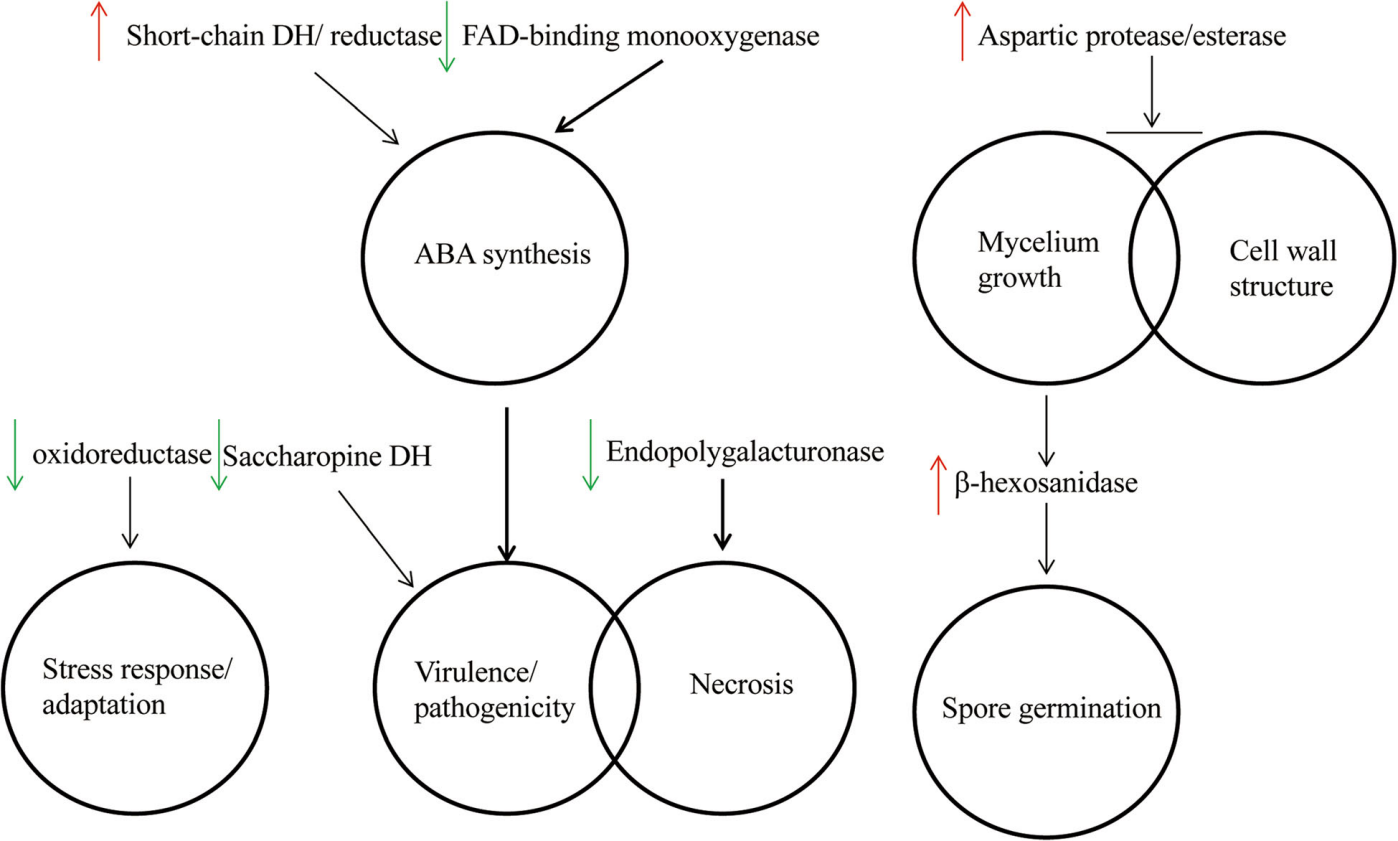
Potential *B. cinerea* functional pathways implicated in the effects of wuyiencin treatment by targeted proteomic analysis. Coloured arrows: indicate either up-regulation or down-regulation following wuyiencin treatment. Black arrows (or red perpendicular symbols) signal enzyme/function relationships validated in *Botrytis cinereal*. ABA: abscisic acid; DH: dehydrogenase

Based on the validated differentially expressed proteins with confirmed *B. cinerea*-associated roles, we formulated a diagram of probable protein-related mechanisms (Fig. 6). Of the up-regulated proteins, aspartic protease inhibits spore germination and growth of *B. cinerea* in addition to efficiently damaging the cell wall [31]. A putative esterase purified from *Botrytis* similarly showed that the protein is up-regulated during glucose depletion and mycelium deterioration, which is likely to signal advanced cell wall destabilization [32, 33]. Another interesting protein identified in this study has previously been reported as a correlate of spore germination inhibition by the biological control agent *Serratia plymuthica* HRO-C48 [34]. While this study did not fully establish the role of the protein, that study combined with our own makes a strong case for the either a direct or indirect role for β-hexosaminidase in the inhibition of spore germination.

The identified protein similar to oxidoreductase has demonstrated a wide range of biological roles in *B. cinerea*. Most relevant to this study is its previously described role as a stress adaptor during metal toxicity challenge [35]. While wuyiencin itself would serve as the stress initiating factor in this instance, the down-regulation of the protein similar to that of the oxidoreductase may signal an attenuation of the stress response by wuyiencin at day 7 following treatment. Two down-regulated proteins identified in our study have previously been shown to be involved in conferring *B. cinerea* virulence and pathogenicity. Saccharopine dehydrogenase is an important enzyme in lysine biosynthesis and is differentially expressed during maturation/spore formation (low abundance during the nascent stages and up-regulated during late apothecium). The protein is also up-regulated by approximately 4-fold during *B. cinerea* plant infection [36]. In *B. cinerea*, endopolygalactourinase is encoded by at least six genes, one of which is required for full virulence [37, 38]. Interestingly, gene knockout studies confirmed that almost all gene products possess necrotizing ability in a range of plant species [38]. Deletion of the gene encoding endopolygalactourinase 2 delayed primary lesion formation by 24 h and reduced lesion expansion by up to 85% in infected tomato and broad bean plants. The wuyiencin-induced reduction in the expression of this virulence-conferring protein observed in this study provides strong evidence that wuyiencin acts either directly or indirectly to silence the pathogenicity of the organism.

Finally, the P450 mono-oxygenase, FAD-binding mono-oxygenase [39] has been shown in *B. cinerea* to play a significant role in abscisic acid (ABA) biosynthesis, a plant hormone involved in negative regulation of defense signaling [40]. In this study, the protein was down-regulated by wuyiencin, indicating that the agent suppressed ABA biosynthesis. However, we unexpectedly found that a short chain dehydrogenase/reductase protein, also involved in ABA synthesis, was simultaneously up-regulated by wuyiencin treatment. Targeted inactivation showed that this protein plays an equally important role in ABA biosynthesis [41]. Currently, we can only reconcile this dichotomy first by understanding that we are still at the very early stages of profiling the proteome and biological pathways of the *Botrytis*-wuyiencin interface and second, by keeping in mind that a majority of previously reported *B. cinerea* and wuyiencin studies have been conducted in infected tissues. While this is true of all proteins profiled in this study, this fact may be all the more relevant in ABA synthesis as the dynamics of this hormone are highly dependent on the host plant where, in fact, *B. cinerea* production of ABA is stimulated by the host plant [42]. Studies have shown that ABA can affect the fungal growth, such as *Aspergillus nidulans* [43], but no references have directly reported the relationship between ABA and sporulation of *B. cinerea,* only a small number of indirect reports have been made in other species, such as *Mohria caffrorum.* The effects of ABA on the germination of spores and growth of protonemata of the fern, *Mohria caffrorum* Sw. revealed ABA did not affect the initial divisions of the spore protoplast leading to the formation of rhizoid and protonema, but inhibited the subsequent elongation of the latter [44]. Previous studies have reported that ABA is related to virulence of *B. cinerea* [45], but recently another perspective based on new findings by Darma et al. are that ABA production may not play a prevailing role in fungal growth or pathogenicity, as demonstrated in *Leptosphaeria maculans* [46], perhaps ABA performs different functions in different species. Thus, interpretation of the role of wuyiencin on ABA production should be taken as a preliminary hypothesis. These speculated relationships of course require further validation but offer logical insights into the possible mechanisms and pathways by which wuyiencin mediates biological control against the pathogenic filamentous *B. cinerea*.

The investigations following this study should focus on validation of the proteins by loss/gain-of-function studies. Similarly, investigations of the effects of wuyiencin on *B. cinerea* at the proteomic level should be expanded to include the host tissue, as the application of wuyiencin will ultimately include that third player. However, the pure focus of the wuyiencin-*Botrytis* relationship in this study provided an opportunity to concentrate solely on the proteins and processes unique to that relationship. Wuyiencin application in field tests has not only corroborated the fungicidal characteristics published previously and echoed by the present study, but has also affirmed the tolerability of organisms such as strawberry and tomato plants. As detailed by the Master’s thesis of Wu, the performance of wuyiencin at 100 ppm is similar to that of the fungicide procymidone, with protective effects lasting up to 8 days in potted plants. In tomato plants, not only was the dose of wuyiencin well tolerated, but actually increased the production of vitamin C and sugar/acid ratio [47]. All these findings indicate warrant the continued investigation and expansion of wuyiencin for agricultural applications and beyond.

## Conclusions

The results of this study confirm the inhibitory properties of the biological control agent wuyiencin on the pathogenic fungus *B. cinerea*. Our findings further elaborate on the proteome of the treated fungus and identify important potential pathways and proteins for further, targeted evaluation. Due to its low toxicity and environmental/agricultural safety, expanding and optimizing the use of wuyiencin without compromising effectiveness or increasing fungicidal resistance could be of great benefit to the agricultural industry and consumers alike. Moreover, enhanced understanding of the protective/antagonistic mechanisms of wuyiencin has a potentially broader applicability, based on its previously demonstrated antimicrobial properties.

## Methods

### Experimental treatment of *Botrytis cinerea* with wuyiencin

The *B. cinerea* strain B05.10 originated from prof. Tudzynski (Münster, Germany) [48] and maintained on potato dextrose agar (PDA) medium at 20 °C under laboratory conditions. It was inoculated on PDA medium [23] containing wuyiencin at a final concentration of 0 ppm (CK), 50 ppm, 100 ppm and 200 ppm. Cultures were incubated at 20 °C and mycelia morphology was observed under a light microscope after 3 days. Conidia were eluted with 0.2% Tween 20 after 7 days and the number of spores in droplets was quantified using a hemocytometer under a microscope. Three replicates were performed for growth measurements and spore production. The final concentration of wuyiencin is approximately 50–100 ppm in agricultural production [6]; this study showed that 200 ppm wuyiencin is lethal to *B. cinerea*, but there is little effect on cell growth at 50 ppm wuyiencin. Thus, we cultivated B05.10 in the dark at 20 °C with 100 ppm and without wuyiencin.

### Scanning electron microscopy analysis

The morphology of the hyphae and their behaviour within the contact zone was investigated by scanning electron microscopy (SEM). After 7 days of cultivation on PDA, B05.10 samples (2 × 4 mm) were fixed in 0.2 M phosphate buffer (with 2% glutaraldehyde, pH 6.8) at 4 °C for 4–6 h and rinsed once for 2 h. The dehydration of samples were treated in a graded acetone series (30, 50, 70, 80, 90, and 100%), with samples immersed in each grade for 30 min and repeated three times for 100% acetone. Finally, the prepared samples were dried using critical point dryer (HCP-2, Hitachi), then coated strain (B05.10) (approximately 200 nm thick) using a sputter coater (S-3400 N, Hitachi). The observation was carried out with a SEM HV (S-3400 N, Hitachi) at 10 kV.

### Transmission electron microscopy

For transmission electron microscopy (TEM) evaluations, B05.10 were cultured on PDA medium for 7 days before samples of hyphae were removed using a clean scalpel and washed three times with sterilized distilled water. Samples were then fixed overnight in 0.1 M phosphate buffer (with 2.5% glutaraldehyde, pH 7.2) at 4 °C. Subsequently, the samples were rinsed with 50 mM phosphate buffer (pH 6.8) three times and post-fixed in 0.1 M cacodylate buffer (with 1% osmium tetroxide, pH 7.0) at 4 °C for 2 h. The dehydrated samples were treated in a gradient ethanol series and embedded in Epon 812 resin. The ultrathin sections were stained in 2% uranium acetate followed by lead citrate. The observation was carried out with a TEM (Hitachi, H-7650) at 80 kV.

### Protein extraction

The standard strain B05.10 of *B. cinerea* was inoculated onto PDA medium containing cellophane at a final concentration of 0 ppm (CK) and 100 ppm of wuyiencin, and placed in a 20 °C incubator. After 7 days, the hyphae were collected. The total proteins of *B. cinerea* were extracted without and with wuyiencin treatment. Three replicates represented control *B. cinerea* and three treatments represented wuyiencin-treated *B. cinerea*.

Hyphae (wet weight 0.5 g) scraped from fresh *B. cinerea* cultured on PDA plates with cellophane were added to 1 ml cold 10% TCA (Trichloroacetic acid)-0.07% β-me/acetone (β-mercaptoethanol). The sample was chilled at 20 °C for at least 1 h before centrifugation at 13,000×g for 30 min at 4 °C. The supernatant was discarded and cold acetone/0.07% β-mercaptoethanol (6 volumes) was added to the pellet. The solution was vortexed vigorously to mix well and stored at 20 °C for 30 min. Sample tubes were inverted several times every 10 min. The lysate was centrifuged twice at 13,000×g for 30 min at 4 °C and each time, the supernatant was discarded. Finally, the *B. cinerea* pellet was air-dried and at room temperature to remove any residual acetone.

Lysis buffer was added to the *B. cinerea* pellet and incubated at room temperature for 30 min. The lysed samples were sonicated at 4 °C according to the following settings: 200 W, 10 s ON/15 s OFF pulses, (repeat 5 times). Finally, sonicated samples were centrifuged at 13,000×g for 30 min at 4 °C. The supernatant was retained and used for further experiments.

### Trypsin digestion of proteins

The total protein was quantified by Bradford method [49]. Samples of protein (200 μg) were placed in a centrifuge tube, mixed evenly with 5 μl DTT (1 M) and incubated for 1 h at 37 °C. After the addition of 20 μl iodoacetamide (IAA, 1 M), the solution was mixed at room temperature for 1 h. Samples were drawn into an ultrafiltration tube and the supernatant was discarded after centrifugation at 14,000×g for 15 min at 4 °C. Subsequently, 100 μl UA (8 M urea, 100 mm Tris-HCL, pH 8.0) were added to the ultrafiltration tube and the supernatant was discarded after two further rounds of centrifugation. TEAB buffer (0.5 M) was added (100 μl) and the supernatant was discarded following another three rounds of centrifugation. Finally, trypsin was added to the ultrafiltration tube at a protein to enzyme ratio of 50:1 and incubated for 12–16 h at 37 °C.

### Tandem mass tag (TMT) labelling

First, 45 μl of TEAB buffer (100 nM) was added to 100 μg re-labelled protein sample, and the total volume made up to 100 μl using distilled water. Next, 5 μl iodoacetamide (175 mM) was added and the sample incubated in the dark for 30 min before the addition of 1 ml pre-chilled acetone and precipitation at − 20 °C overnight. After centrifugation at 12,000×*g*, the protein precipitate was air-dried and dissolved in 100 μl TEAB buffer (100 mM). The tryptic digests were then desalted using Pierce C18 spin columns (Thermo Scientific) according to manufacturer’s instructions. The digested peptides were labelled using a TMT kit according to the manufacturer’s instructions. After incubation for 1 h at room temperature, the labeling reaction was terminated by incubation with 5% hydroxylamine for 15 min. All samples were then combined and stored at − 80 °C.

### Ion exchange chromatography

Off-line grading of labeled peptides was performed using the Waters HPLC system (series 2695) and the strong cation exchange column PolyLC Polysulfoethyl aspartamide column (100 mm × 2.1 mm, 5 μm particle size, 300 A pore size; PolyLC, Columbia, MD, USA). For gradient elution, 100% Buffer A (10 mM potassium dihydrogen phosphate, 15% acetonitrile, pH 2.7) was switched to 100% Buffer B (10 mM potassium dihydrogen phosphate, 15% acetonitrile, 500 mM potassium chloride, pH 2.7) over a period of 40 min. Gradient elution was performed at a flow rate of 200 μl/min, and elution peak monitoring and collection was performed at 220 nm using a Waters 2998 PDA module.

### LTQ Orbitrap mass spectrometry

The fractionated peptides were separated by reverse phase liquid chromatography (RP-LC) using an Easy-nLC 1000 nanoflow high performance liquid chromatography system (Thermo Fisher Scientific, Odense, Denmark) with a capillary C18 reverse phase column (Acclaim PepMap, NanoViper C18, 50 μm × 15 cm). For linear gradient elution (5–40%) elution, Buffer A (0.1% formic acid in water) was switched to Buffer B (0.1% formic acid, 98% acetonitrile) over a period of 60 min, at a flow rate of 300 nl/min. The separated components entered the LTQ Orbitrap combined mass spectrometer (Thermo Scientific) directly for tandem mass spectrometry identification using the default optimization parameters of the instrument, which were automatically adjusted according to the specific operating conditions. Level 1 scan range was m/z 350–1800, and the 10 ions with the highest intensity were selected for secondary ion trap analysis. Mass spectrometry data were downloaded to a local computer for analysis using Proteome Discoverer™ 1.3 software (Thermo Fisher Scientific).

### Bioinformatics analysis

Proteome Discoverer™ 1.3 software (Thermo Fisher Scientific) was used in combination with the grey mould database uniprot-botrytis_organism_Acinerea. For relative quantitation of identified proteins, the proteins required a “high” peptide confidence interval and a maximum of two missed trypsin cleavages. N-term acetylation and oxidation (M) were set as variable modifications and carbamidomethylation and oxidation (C) as a fixed modification. The search was performed with a peptide mass tolerance of 15 ppm and a product ion tolerance of 0.02 Da with a 1% false discover rate (FDR). Differential protein screening (determined by the ratio in the treated samples and their corresponding untreated controls) was performed under the condition of 1.5 difference multiples (fc = fold change) and *P* < 0.05 threshold and under the condition of 2.0 difference multiples.

### Protein hierarchical cluster analysis

In this study, we used hierarchical clustering analysis, in which each sample is first considered individually as a class, and the distances between the different classes are merged, before recalculation of the distance between classes. This process continues until all samples are grouped into one category. The standard for calculating the cluster distance indicator d (distance) uses the Euclidean distance, which represents the distance between classes using the full join method, also known as the longest distance method. The pair-wise ‘distance’ of proteins between the control (CK) and wuyiencin-treated (100 ppm) samples was subjected to Log2 normalisation and heatmaps and dendrograms were subsequently generated by pHEATMAP (R package) and g-plots, respectively. All statistical analyses were performed in the R environment.

### GO enrichment analysis, functional classification, and expression profile analysis

For GO annotation (http://www.geneontology.org), the GO database (http://www.geneontology.org/page/download-go-annotations) is used to classify proteins into cellular components, molecular function, and biological process categories. Functional annotation of proteins was performed in this study by BLAST2GO, a comprehensive suite for functional analysis, in conjunction with the Perl computer programming language. Annotation was based on sequence similarity identified through the hypergeometric test, where *P* < 0.05 was considered to be significant. Significant differences in the expression of proteins in the treatment group compared with those in the control group were identified by *t*-tests. Blast results were mapped to uniprot-botrytis_organism_Acinerea and functional classification of differentially expressed proteins was performed using the online GO classification software QuickGo (http://www.ebi.ac.uk/QuickGO/). In-depth analysis of the main metabolic pathways was performed by KEGG pathway annotations generated from the mapping of GO terms to their enzyme code equivalents, according to the KEGG database (http://www.kegg.jp/kegg/pathway.html).

### Target analysis by parallel reaction monitoring (PRM)

Q-Exactive HF mass spectrometer was used for PRM analysis (Thermo Fisher Scientific). The 90 min LC gradient settings was carried out at the Beijing Bangfei Bioscience Co., Ltd. (Beijing, China). The MS acquisition mode contained both a full and a time-scheduled scan. The full scan was taken at a resolution of 120,000 at m/z 200 (scan mass range of 300 to 1300 m/z) with a target AGC of 3e^6^ and maximum injection fill time of 80 ms. The scheduled scan was carried out at a resolution of 15,000 at m/z 120 with a target AGC of 2e^5^ and maximum injection fill time of 45 ms. The precursor ion of target peptides was isolated with a 2 Da window (elution time set as ±2.5 min).

### PRM MS analysis

MS raw data acquired from the target proteomics analysis were searched with SEQUEST integrated in Proteome Discoverer (version 1.4, Thermo Fisher Scientific, San Jose, CA, USA) against the uniprot-botrytis_organism_Acinerea database. Peptide probabilities were calculated by the Percolator algorithm in Proteome Discoverer, with a FDR set to 0.01. The generated .msf file was used to create a library in Skyline (version 3.1.0) and the cut-off score was set as 0.90. The target precursors list was then uploaded to Skyline and the six most intense product ions matching the library were selected as transitions. Peak extraction and manual inspection of fragment ion mass was performed and corrected according to the transitions, retention time, mass accuracy and MS/MS spectra. Each precursor was assigned the six most intense transitions and only transitions shared by all of the samples were used for quantification. The peak area of each transition was extracted and the area under curve (AUC) was extracted and exported to MSstats for further data analysis.

## Data Availability

The mass spectrometry proteomics data have been deposited to the ProteomeXchange Consortium (http://proteomecentral.proteomexchange.org) via the PRIDE partner repository with the dataset identifier < px-submission #381075>. All data generated or analyzed during this study are included in this manuscript (and its supplementary information files). All authors read and approved the final manuscript.

## Abbreviations

ABA: Abscisic acid
cAMP: Cyclic Adenosine monophosphate
FAD: Flavin adenine dinucleotide
iTRAQ: Isobaric tags for relative or absolute quantitation
MAP: Mitogen-activated protein
PRM: Parallel reaction monitoring
